# Bibliometric analysis of studies of the Arctic and Antarctic polynya

**DOI:** 10.3389/frma.2023.1100845

**Published:** 2023-03-16

**Authors:** Tianyu Zhang, Haiyi Ren, Mohammed Shokr, Fengming Hui, Xiao Cheng

**Affiliations:** ^1^School of Geospatial Engineering and Science, Sun Yat-sen University, and Southern Marine Science and Engineering Guangdong Laboratory (Zhuhai), Zhuhai, China; ^2^State Key Laboratory of Remote Sensing Science, College of Global Change and Earth System Science, Beijing Normal University, Beijing, China; ^3^Science and Technology Branch, Environment and Climate Change Canada, Toronto, ON, Canada

**Keywords:** polynya oceanography, Arctic ocean, Antarctic, sea ice, research trends

## Abstract

Based on the polar polynya-related 1,677 publications derived from the Web of Science from 1980 to 2021, this study analyses the scientific performance of polar polynya research with respect to publication outputs, scientific categories, journals, productive countries and partnerships, co-cited references, bibliographic documents and the thermal trends of keywords. The number of publications and citations on polar polynya has increased 17.28 and 11.22% annually since the 1990s, respectively, and those numbers for Antarctic polynya have surpassed that of the Arctic polynya since 2014. Oceanography, geosciences multidisciplinary, and environmental sciences were the top 3 scientific categories in the Arctic and Antarctic polynya research field. Nevertheless, ecology and meteorology are gaining ground in the Arctic and the Antarctic recently. The Journal of Geophysical Research-Oceans accommodated most publications for both polar regions, followed by Deep-Sea Research Part II-Topical Studies in Oceanography and Polar Biology. The Continental Shelf Research and Ocean Modeling were favored journals in Arctic and Antarctic polynya research, respectively. The USA dominated the polar polynya study field with 31.74%/43.60% publications on the Arctic/Antarctic polynya research, followed by Canada (40.23%/4.32%) and Germany (17.21%/11.22%). Besides, Australia occupied the second most popular position in the Antarctic polynya research. The keywords analysis concluded that the polynya topics that generated the most interest were altered from model to climate change in the Arctic and ocean water and glacier in the Antarctic over time. This study gives a summary of the polar polynya scientific field through bibliometric analysis which may provide reference for future research.

## 1. Introduction

Polynyas are recurrent areas that are predominantly or entirely features ice-free in an area where otherwise should be ice-covered because of the freezing atmospheric temperature (Smith et al., 1990). They occur at several locations in the Arctic and numerous locations, mostly coastal, around the Antarctic. They hold ecological importance due to their high primary productivity (La et al., 2019; Von Berg et al., 2020) and provision of habitats for polar-living mammals and birds (Heide-Jørgensen et al., 2013; Labrousse et al., 2018). Polynyas have an overall area of tens to tens of thousands of square kilometers and a duration of several days to weeks (Barber and Massom, 2007). In the current context of global climate change, the natural regions of polynyas, which are too small to be studied directly have received attention as a possible early indicator of the effects of local warming at the polar regions and as a potential impact of global warming at high latitudes (Stewart et al., 2019; Lockwood et al., 2021; Monroe et al., 2021). The environmental significance of the polynya is manifested in a few aspects: (1) high ice production (Preußer et al., 2019; Dai et al., 2020; Nakata et al., 2021), (2) significant heat flux from the ocean to the atmosphere (Haid and Timmermann, 2013; Ohshima et al., 2020), (3) salt flux into the ocean (Signorini and Cavalieri, 2002; Durski and Kurapov, 2020), (4) sea ice export (Kern, 2008; Bruneau et al., 2021), and (5) the polar climate and the halocline maintenance of Arctic (Bauch et al., 2012; Hirano et al., 2018). Additionally, polynyas are regarded as probable locations for formation of Antarctic deep water (Hirano et al., 2018; Ryan et al., 2020).

In the context of the rapidly changing polar environment (Shepherd et al., 2018; Landrum and Holland, 2020; Myers-Smith et al., 2020), considerable attention has been given to the Arctic and Antarctic polynyas in the aforementioned scientific fields. Several literature reviews have explored the scientific development of polar polynya observation and modeling, dense water formation, and sea ice production (Morales Maqueda et al., 2004; Ohshima et al., 2016). Smith et al. (1990) made a very informative overview of the physical processes and environment of polynyas. The first comprehensive, multidisciplinary look at polynyas and their interactions with the ocean, atmosphere and biology was given in Smith and Barber (2007). However, a bibliometric study that organizes the production in this field is still missing.

This paper seeks to provide a bibliometric approach to polynya research related to the advancement from 1980s till 2021 in both the Arctic and Antarctic, from the aspect of publication outputs, scientific terms, published journals, international collaboration, co-cited references, bibliographic documents and the development of keywords. The main objectives of this study are to reveal the scientific progress and categorical and national patterns of Arctic and Antarctic polynya studies and to identify the state of the art in this field.

## 2. Materials and methods

### 2.1. Materials

Our bibliometric data is built based on publications searched from the Web of Science (WoS) core collection. In the scientific community, polynya is also spelt polynia, and their plural forms are commonly used. Therefore, the query phrase of [ALL = (polynya or polynyas or polynia or polynias) AND LA = (“ENGLISH”)] (ALL means all fields, LA means language) is used for retrieving publications in the WoS core collection with time limited between 1980-01-01 and 2021-12-31 and language restricted to English. This primary search strategy yielded 1,899 publications. Furthermore, a truncation using [TS = (Polynia^*^) OR TS = (Polynya^*^)] AND LA = (“English”) yielded 1,885 publications, which was very close to the primary search.

Excluding irrelevant publications is a key process in bibliometric analysis (Donthu et al., 2021). Because the original goal of this study was to explore studies of polynya occurring in the polar regions, those publications that focused on polynya occurring in the Gulf of Alaska, the Baltic Sea, the Sea of Japan, lagoons, and inland lakes were manually filtered out. In addition, publications with only one or two occurrences of the search terms in the reference section were also screened. After the above search and filter strategies, 1,755 publications spreading over 11 document types are obtained with the top three types in article (1,530, 87.18%), proceeding paper (147, 8.38%), and review (61, 3.48%). The first two types, out of a total of 1,677, are used in this study for investigation. For further analysis, the 1,677 publications are divided manually into Arctic (869 publications) and Antarctic (702 publications) categories. The rest 106 publications, which mainly focus on methodology or model research are treated as “other” category. Full records and citation information for each publication were derived from the WoS website to form the analysis database, including authors, titles, sources, abstracts, keywords, cited references, and funding records. Each journal covered by the WoS Core Collection is assigned one or more WoS categories (WoSc) from its 254 scientific categories. Those scientific categories are used in the analysis of scientific terms. Besides, the rank and quartile Journal Impact Factor (JIF) of each WoSc for each journal are obtained from the 2021 Journal Citation Reports provide by Clarivate.

### 2.2. Methods

We use some bibliometric analysis parameters as adopted by Ji et al. (2014) and further derived parameters and their variants for the Arctic and Antarctic (e.g., TP_N_, TP_S_). The abbreviation and explanation of those parameters are summarized in Table 1. The basic parameters consist of the total number of publications (TP), citations (TC), cited number of references (NR), authors (AU), independent publications (IP), and international collaboration publications (ICP). Further derived parameters, like the average citations (TC/TP), cited references (NR/TP) and authors per article (AU/TP), are used to quantify the publication outputs performances, the major scientific categories and journals. The ratio parameter of ICP/TP is used to describe the proportion of international collaboration articles. Based on the records of 1,677 publications, the above parameters were calculated for each year, journal, scientific category, and country. The Arctic and Antarctic polynya publications were quantified separately.

**Table 1:** The abbreviation and explanation of bibliometric indicators used in this study.

**Abbreviation**	**Explanation**	**Variants**
TP	The total number of publications.	TP_N_, TP_S._
TC	The total number of citations.	TC_N_, TC_S_
NR	The total number of cited number of references.	NR_N_, NR_S_
AU	The total number of authors.	AU_N_, AU_S_
IP	The total number of independent publications, whose authors are from the same country.	\
ICP	The total number of international collaboration publications, which consist of authors from more than one country or region.	ICP_N_, ICP_S_
TC/TP	The average citations per article (the slash means division).	TC_N_/TP_N_, TC_S_/TP_S_
NR/TP	The average cited references per article.	NR_N_/TP_N_, NR_S_/TP_S_
AU/TP	The average authors per article.	AU_N_/TP_N_, AU_S_/TP_S_
ICP/TP	The proportion of international collaboration articles (in the unit of %).	ICP_N_/TP_N_, ICP_S_/TP_S_
WoSc	The number of Web of Science category	\

The subscripts N and S denote Arctic and Antarctic, respectively.

To assess the relative vitality of research in the polar polynya domain in comparison to other research subjects in the Arctic and (or) Antarctic domains, comparisons are made, denoted by the proportion of research in the polynya domain to all research in the Arctic and (or) Antarctic domains. All publications related to both polar regions, the Arctic and Antarctic, were obtained with the search query phases of ALL = (Arctic or Antarctic), ALL = (Arctic), and ALL = (Antarctic), respectively.

The full records of all 1,677 publications were organized into a uniform format and imported to the MATLAB software to perform the following processes *via* coding. For the international collaboration analysis, authors from England, Scotland, Northern Ireland, and Wales were combined into the United Kingdom (UK).

Prior to the evolution analysis based on keywords, the keyword list requires some additional refinements and normalization (Muñoz-Écija et al., 2022). Publications that did not have author keywords or keywords plus were identified and excluded. Keywords in the singular or plural, e.g., “polynya” and “polynyas,” as well as those that differ only in a connector, e.g., “sea ice” and “sea-ice,” were identified and treated as the same. All words were converted to lowercase letters. No abbreviation or acronyms issues in this study. The gaps in the list of author keywords were filled using the keywords plus, which were provided by WoS, rather than combining them. Most of the resulting graphs are drawn using MATLAB.

The VosViewer software (van Eck and Waltman, 2010) was used to assist in the co-citation analysis of references and authors, the bibliographic coupling analysis of documents, the presentation of scientific networks between different countries, and the visualizations of keyword clustering and tendency in the field of polynya research. The method of fractional counting was adopted. For reference assessment, a minimum number of citations of 20 was adopted. In terms of keywords analysis, a minimum occurrence threshold of 5 was used (Muñoz-Écija et al., 2017). In all the cluster maps in the results section deduced from VosViewer, the color indicates clusters, the circle size indicates the number of occurrences, the thickness of the lines indicates the strength of linkage, and the distance between circles indicates their relationship. In the tendency map, color denotes time. Collaboration relationships between countries, derived from the online bibliometric platform (http://bibliometric.com/), were also used to assist the international collaboration analysis (**Figures 5C**, **D**).

## 3. Results

### 3.1. Basic characteristics of publications

**Figure 1:**
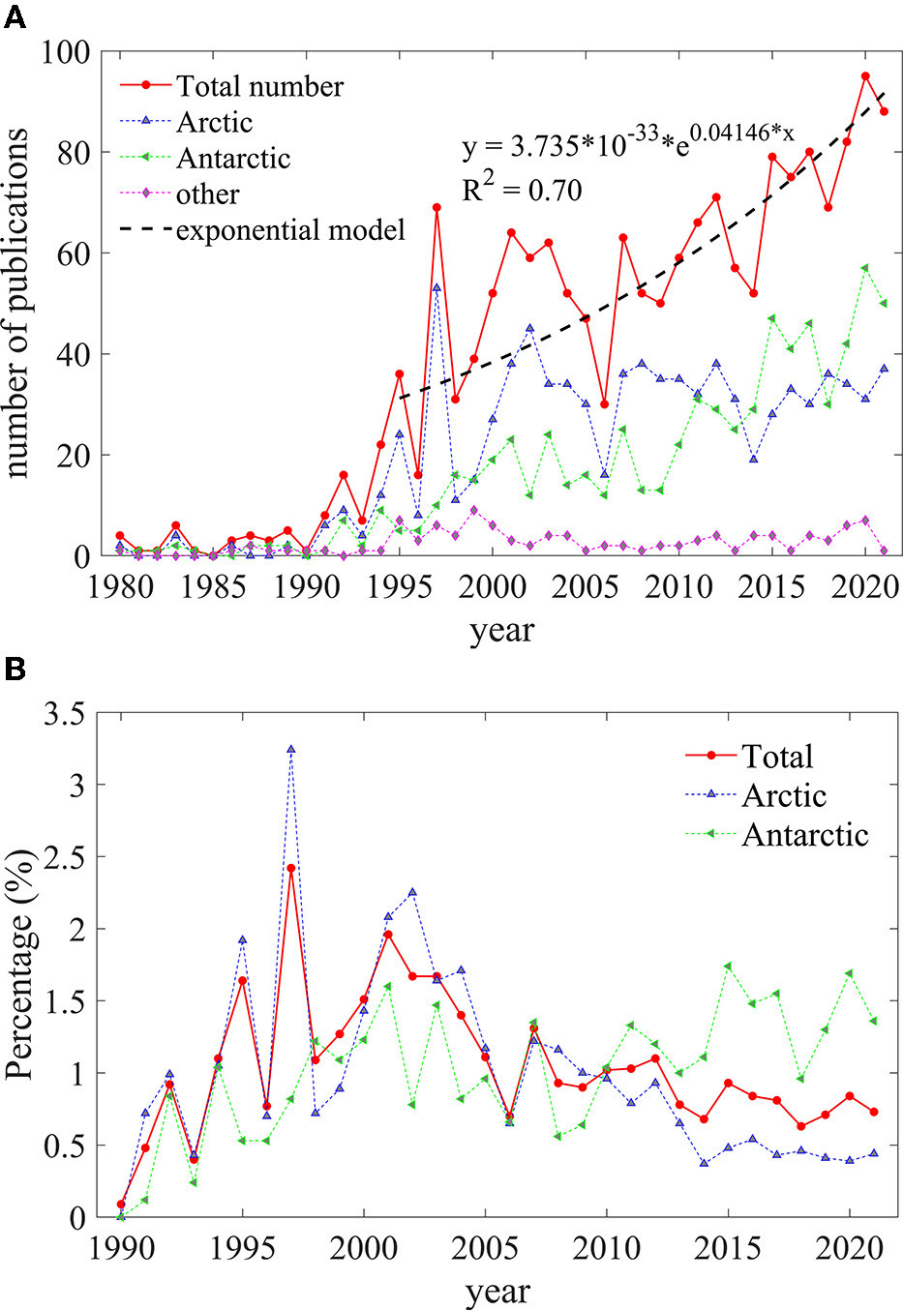
**(A)** Quantitative distribution and **(B)** occupation trends of publications about polynya for both polar regions, the Arctic, and Antarctica, respectively, 1980–2021.

Figure 1A shows the trend of the number of publications in polar polynya studies. The trend shows a rapid but fluctuating increase since around 1990, from one publication in 1990 to 88 publications in 2021. The exponential tendency line (black dotted line) confirms the growth trend of publications. The annual increase of publication number (rate) for both polar, Arctic, and Antarctic polynya research are 2.44 (17.28%), 0.93 (28.37%), and 1.52 (15.30%), respectively, from 1995 to 2021. The larger number of publications on Arctic polynya in 1997 is attributed to a specific issue of the Journal of Marine Systems in Northeast Water polynya based on an international symposium held in 1995 (Hirche and Deming, 1997). In the recent decade, the increasing trend in the Antarctic is 3.08 (16.34%), which surpassed the 0.17 (2.85%) of the Arctic. There is no clear trend in the study of the “other” category.

Figure 1B gives temporal percentage statistics of polynya researches with respect to publications of all subjects related to both polar regions, Arctic and Antarctic. Though the increasing trend is partly attributed to additional publications recorded by Science Citation Index (SCI), the ratio of polynya publications for both polar regions within the SCI publications has decreased from 1.9% in the 2000s to 0.70% in the 2020s (red line). The percentage of Arctic polynya with respect to the Arctic-related publications declined more steeply (blue line). On the contrary, the ratio of Antarctic polynya research shows a slightly increasing tendency in the recent 10 years with a larger rate than Arctic (green line in Figure 1B). In short, the Antarctic polynya, which is of growing interest to the scientific community, will predictably receive more publications in the future than the Arctic polynya.

The temporal characteristics of references, citations, and authors are given in Table 2 for both polar regions, Arctic, and Antarctic, respectively, from 1995 to 2021. The average number of references for both polar regions (NR/TP), the Arctic (NR_N_/TP_N_) and the Antarctic (NR_S_/TP_S_) shows significant growth from 38.22, 41.88, and 37.60 in 1995 to 69.39, 74.14, and 66.56 in 2021, respectively, which implies that the knowledge of polar polynya research is expanding. The average citations ranged from 35.12 to 36.87 for the three categories (TC/TP, TC_N_/TP_N_, and TC_S_/TP_S_). In addition, the increasing average number of authors (AU/TP), from less than three in 1995 to over six in 2021, indicates higher scholarly participation and a growing polar polynya research community.

**Table 2:** Basic scientific descriptions of average references (NR/TP), citations (TC/TP), and authors (AU/TP) per publication of polynya research from 1995 to 2021, for both polar, Arctic (N) and Antarctic (S), respectively.

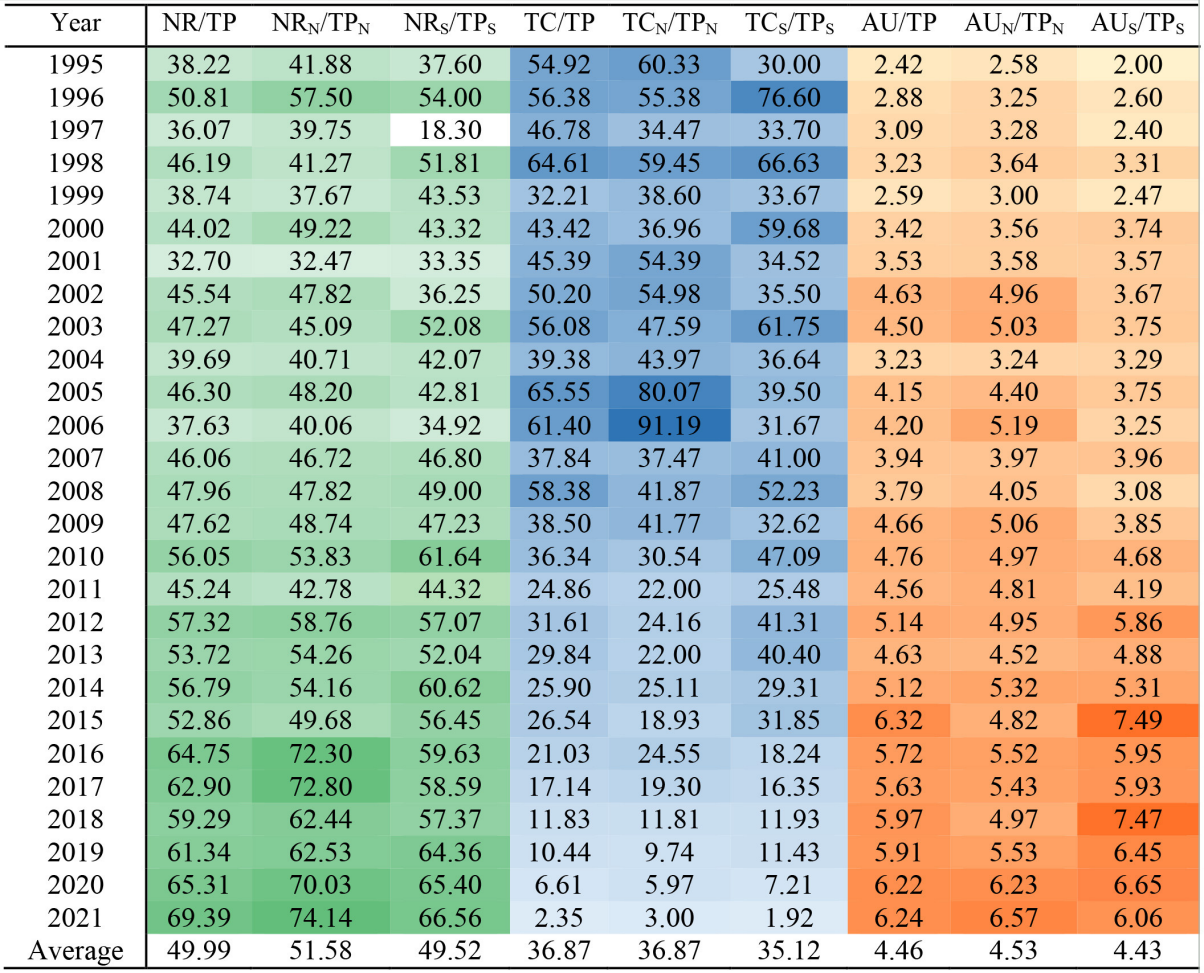

The dark color indicates high value, and vice versa.

### 3.2. Scientific categories and journals

**Figure 2:**
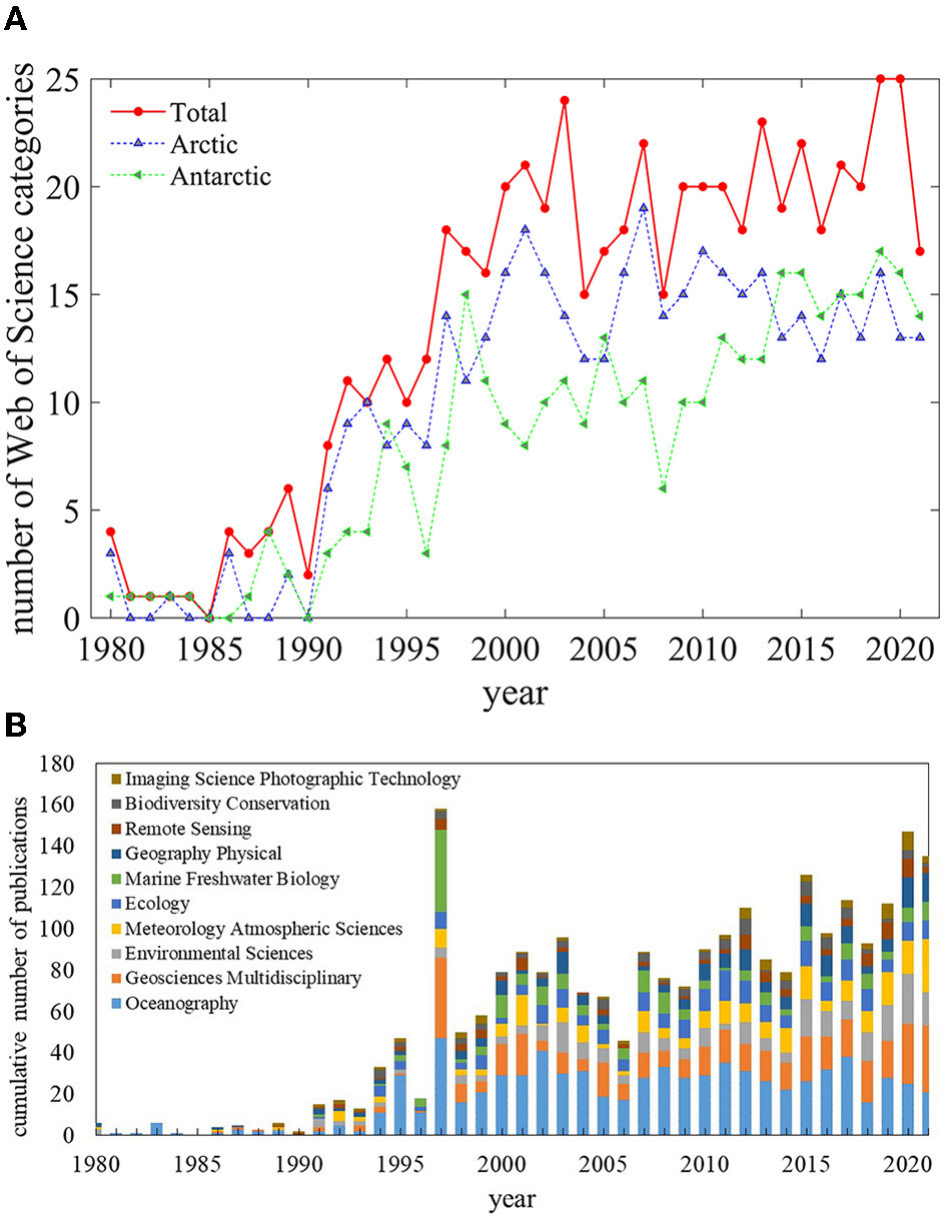
**(A)** The number of Web of Science categories on polynya research for both polar, the Arctic and the Antarctic polynya research, respectively, 1980–2021. **(B)** Cumulative publications number of the top 10 productive WoS categories on polar polynya research, 1980–2021.

The 1,677 publications on polar polynya studies cover 61 WoS categories, with 47 for the Arctic and 41 for the Antarctic. The annual number of WoS categories in Figure 2A shows a tendency of WoS categories to grow from < 10 in the 1980s to more than 20 in the 2020s, indicating an increasing subject coverage for polynya studies.

Figure 2B gives a temporal evolution in the cumulative top 10 most productive polynya research categories for both polar regions from 1980 to 2021, namely oceanography (occupied 46.21% of all publications), geosciences multidisciplinary (14.43%), environmental sciences (14.4%), meteorology atmospheric sciences (14.07%), ecology (12.88%), marine freshwater biology (11.33%), geography physical (10.14%), remote sensing (5.90%), biodiversity conservation (5.25%) and imaging science photographic technology (4.47%). Figure 2B shows the dominance categories of oceanography over the last 40 years in both polar regions, with the remaining categories characterized by frequent shifts in ranks. The striking year of 1997 with the highest cumulative number of publications from the top 10 categories resulted from its high productivity, as shown in Figure 1A.

Table 3 gives an overview of the top 10 WoS categories, in terms of the number of publications, for the Arctic and Antarctic regions separately from 1980 to 2021. The two scientific categories, oceanography and geosciences multidisciplinary, dominate polar polynya studies. The environmental sciences and meteorology atmospheric sciences are also crucial to both polar polynya research, with more than 90 publications. The three categories of ecology, marine freshwater biology, and biodiversity conservation occupy a more prominent place in Arctic polynya research than in Antarctic. For the study of Antarctic polynya, the most five significant categories are of meteorology atmospheric sciences, environmental sciences, physical geography, remote sensing, and imaging science photographic technology. The number of publications on marine freshwater biology, ecology, and biodiversity conservation associated with the Antarctic polynya is 69.66%, 59.48%, and 59.06%. These are less than those associated with the Arctic polynya. In contrast, there are more publications on meteorology atmospheric sciences (+29.79%), imaging science photographic technology (+27.59%), and remote sensing (+6.98%) associated with Antarctic polynya than in the Arctic.

**Table 3:** Top 10 WoSc on the Arctic and Antarctic polynya research, respectively, from 1980 to 2021.

**WoSc**	**TP_N_**	**% of 869**	**TP_S_**	**% of 702**	**Ratio (%)**	
Oceanography	418	48.18	305	43.45	−27.03	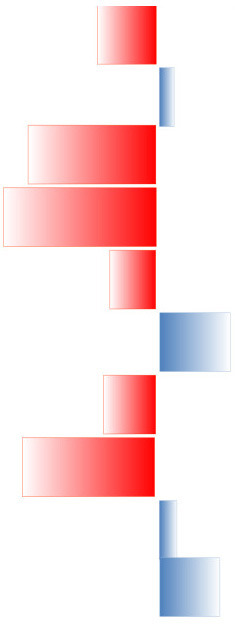
Geosciences multidisciplinary	189	21.75	197	28.06	+4.23	
Ecology	153	17.61	62	8.83	−59.48	
Marine freshwater biology	145	16.69	44	6.27	−69.66	
Environmental sciences	131	15.07	104	14.81	−20.61	
Meteorology atmospheric sciences	94	10.82	122	17.38	+29.79	
Geography physical	90	10.36	70	9.97	−22.22	
Biodiversity Conservation	62	7.13	26	3.70	−59.06	
Remote sensing	43	4.95	46	6.55	+6.98	
Imaging science photographic technology	29	3.34	37	5.27	+27.59	

Ratio equals to (TP_*S*_- TP_*N*_)/TP_*N*_. Red and blue data bars denote negative and positive ratios, respectively. The dark color indicates high value, and vice versa.

The temporal evaluation of the top 15 WoS categories over the last three decades for the Arctic and Antarctic polynya studies is given in Table 4, respectively. The largest deviation has contributed to the rapid decline (−11.19%) in research into marine freshwater biology in the Arctic. This was followed by a downward trend of −9.95 and −3.20% for the Arctic and Antarctic oceanographic polynya studies, respectively. However, the number of publications devoted to the Antarctic oceanographic polynya research has increased, and since about 2014 there have been more oceanographic polynya studies in the Antarctic than in the Arctic. On the other hand, the top three increasing categories from 1990 to 2020 for Arctic polynya studies are environmental sciences (+6.46%), geography physical (+4.41%) and ecology (+3.93%). The corresponding categories for the Antarctic polynya are multidisciplinary sciences (+2.10%), Geosciences Multidisciplinary (+2.02%) and environmental sciences (+1.74%). In particular, the categories of imaging science photographic technology, remote sensing and multidisciplinary sciences on both Arctic and Antarctic polynya studies have shown relatively higher growth over the last decade. At the same time, Antarctic polynya research is characterized by a rapidly increasing number of publications in meteorology atmospheric sciences, and Arctic polynya study is featured with more research on geosciences multidisciplinary. These variations also provide insight into the changing topic of polar polynya research.

**Table 4:** The top 15 frequent WoSc assemblies from 1991 to 2020 for the Arctic and Antarctic polynya research.

**WoSc_N_**	**Rank for Arctic (count, %)**			**WoSc_S_**	**Rank for Antarctic (count, %)**		
	**1991–2000**	**2001–2010**	**2011–2020**		**1991–2000**	**2001–2010**	**2011–2020**
Oceanography↓↓	1 (106, 30.64)	1 (181, 31.42)	1 (115, 20.68)	Oceanography↑↓	1 (42, 28.57)	1 (90, 30.00)	1 (154, 25.37)
Geosciences multidisciplinary↑↓	3 (55, 15.90)	4 (50, 8.68)	2 (74, 13.31)	Geosciences multidisciplinary↑↑	2 (21, 14.29)	2 (54, 18.00)	2 (99, 16.31)
Ecology↑↑	4 (25, 7.23)	2 (58, 10.07)	3 (62, 11.15)	Meteorology atmospheric sciences↑↑	3 (14, 9.52)	5 (20, 6.67)	3 (66, 10.87)
Environmental sciences↑↑	5 (15, 4.34)	5 (47, 8.16)	4 (60, 10.79)	Environmental sciences↑↑	4 (11, 7.48)	3 (28, 9.33)	4 (56, 9.23)
Geography physical↑↑	6 (14, 4.05)	6 (22, 3.82)	5 (47, 8.45)	Ecology↑↑	6 (8, 5.44)	6 (19, 6.33)	5 (34, 5.60)
Meteorology atmospheric sciences↑↑	7 (13, 3.76)	7 (34, 5.90)	6 (40, 7.19)	Geography physical↑↓	4 (11, 7.48)	4 (22, 7.33)	6 (28, 4.61)
Marine freshwater biology↓↓	2 (58, 16.76)	3 (51, 8.85)	7 (31, 5.58)	Remote sensing↑↑	8 (6, 4.08)	8 (10, 3.33)	6 (28, 4.61)
Biodiversity conservation↑↑	8 (12, 3.47)	8 (20, 3.47)	8 (28, 5.04)	Imaging science photographic technology↑↑	10 (4, 2.72)	10 (6, 2.00)	8 (25, 4.12)
Remote sensing↑↑	9 (11, 3.18)	9 (11, 1.91)	9 (20, 3.60)	Multidisciplinary sciences↑↑	12 (2, 1.36)	19 (1, 0.33)	9 (21, 3.46)
Imaging science photographic technology↑↑	10 (7, 2.02)	15 (5, 0.87)	10 (16, 2.88)	Marine freshwater biology↑↓	6 (8, 5.44)	7 (13, 4.33)	10 (19, 3.13)
Multidisciplinary sciences↑↑	-	21 (3, 0.52)	11 (12, 2.16)	Biodiversity conservation↑↓	8 (6, 4.08)	11 (4, 1.33)	11 (16, 2.64)
Engineering electrical electronic↑↑	18 (1, 0.29)	14 (5, 0.87)	12 (10, 1.80)	Chemistry multidisciplinary↑↑	–	–	12 (11, 1.81)
Engineering environmental↑↑	27 (1, 0.29)	10 (9, 1.56)	13 (9, 1.62)	Microbiology↑↑	12 (2, 1.36)	14 (2, 0.67)	13 (9, 1.48)
Geochemistry geophysics↑↑	17 (1, 0.29)	11 (7, 1.22)	14 (7, 1.26)	Geochemistry geophysics↑↓	11 (3, 2.04)	9 (7, 2.33)	14 (7, 1.15)
Anthropology↑↑	–	–	15 (4, 0.72)	Engineering electrical electronic↑↑	18 (1, 0.68)	11 (4, 1.33)	15 (5, 0.82)

The arrow indicates trend in publication number (the first) and variation tendency (the second). Up arrow means increasing and down means decreasing.

**Figure 3:**
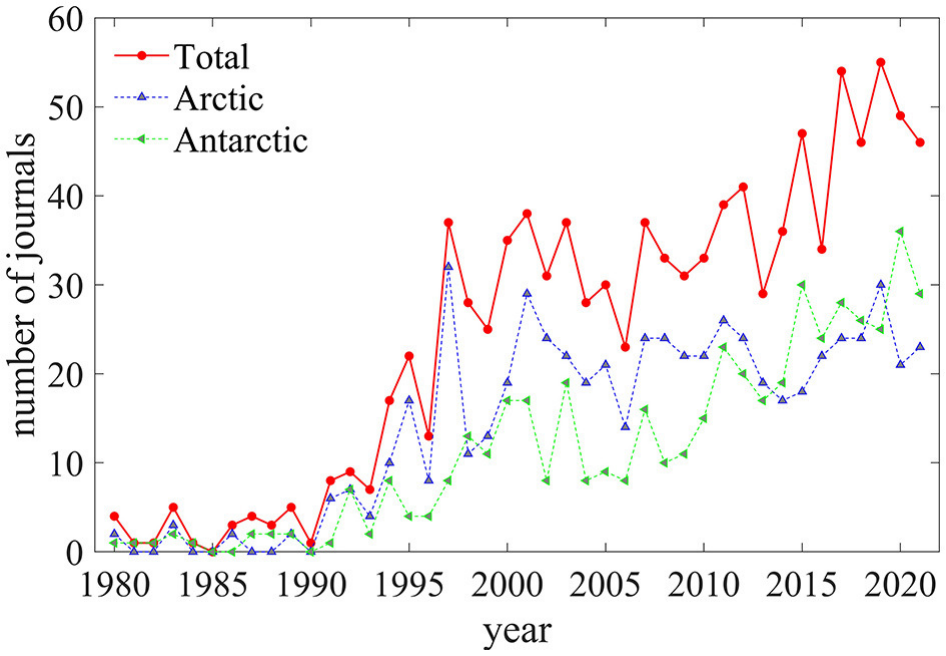
The number of journals on polynya research for both polar, the Arctic and the Antarctic polynya research, respectively, 1980–2021.

Between 1980 and 2021, there were 277 journals published research about polar polynya with an increasing trend (Figure 3). The average number of journals devoted to Arctic and Antarctic polynya studies have reached 24 and 29 in the last 5 years, respectively. The year 2007 also showed a steep increase in journal numbers. The top 16 journals (7.22%) which occupied approximately one third of publications (30.95%) and citations (35.92%) can be regarded as core journals related to polar polynya research (Table 5).

**Table 5:** The citation characteristics of top 16 journals on polar polynya research from 1980 to 2021 for publications in both polar, Arctic and Antarctic, with journal's Web of Science categories (WoSc), JIF rank and quartile and relative rank in the brackets.

**Journal**	**WoSc (position)**	**JIF rank, quartile**	**TP (rank)**	**TC/TP**	**TP_N_ (rank)**	**TC_N_/TP_N_**	**TP_S_ (rank)**	**TC_S_/TP_S_**
Journal of Geophysical Research-Oceans	Oceanography	12/66, Q1	267 (1)	46.10	129 (1)	43.05	114 (1)	23.51
Deep-Sea Research Part II-Topical Studies in Oceanography	Oceanography	24/66, Q1	100 (2)	53.14	52 (3)	59.52	47 (2)	40.19
Polar Biology	Biodiversity conservation Ecology	34/65, Q3 113/173, Q3	76 (3)	25.87	56 (2)	28.11	20 (6)	26.30
Journal of Marine Systems	Geosciences, multidisciplinary Marine and freshwater biology Oceanography	100/201, Q2 31/113, Q1 22/66, Q2	65 (4)	34.77	50 (4)	36.78	16 (9)	33.31
Geophysical Research Letters	Geosciences, multidisciplinary	25/201, Q1	63 (5)	29.16	22 (8)	34.59	38 (3)	27.47
Marine Ecology Progress Series	Ecology Marine and Freshwater Biology Oceanography	88/173, Q3 34/113, Q2 23/66, Q2	50 (6)	52.46	42 (5)	50.48	7 (20)	25.86
Journal of Physical Oceanography	Oceanography	15/66, Q1	40 (7)	31.45	10 (17)	33.5	17 (8)	28.41
Cryosphere	Geography, physical Geosciences, multidisciplinary	5/48, Q1 22/201, Q1	33 (8)	18.91	16 (11)	22.19	13 (12)	33.00
Antarctic Science	Environmental sciences Geography, physical Geosciences, multidisciplinary	221/279, Q4 33/48, Q3 143/201, Q3	33 (8)	26.79	\	\	33 (4)	58.52
Arctic	Environmental sciences Geography, physical	245/279, Q4 37/48, Q4	32 (10)	26.91	31 (6)	26.26	\	\
Deep-Sea Research Part I-Oceanographic Research Papers	Oceanography	21/66, Q2	31 (11)	44.81	12 (12)	62.58	19 (7)	23.11
Journal of Climate	Meteorology and atmospheric sciences	20/94, Q1	27 (12)	27.85	4 (42)	22.50	21 (5)	33.19
Atmosphere-Ocean	Meteorology and atmospheric sciences oceanography	81/94, Q4 43/66, Q3	26 (13)	28.23	23 (7)	27.22	3 (47)	66.33
Ocean Modeling	Meteorology and atmospheric sciences oceanography	56/94, Q3 18/66, Q2	25 (14)	16.40	5 (30)	9.40	16 (10)	43.56
Continental Shelf Research	Oceanography	29/66, Q2	25 (14)	36.24	17 (9)	42.88	8 (18)	37.25
Remote Sensing	Environmental sciences Geosciences, multidisciplinary Imaging science and photographic technology remote sensing	83/279, Q2 30/201, Q1 6/28, Q1 11/34, Q2	21 (16)	8.05	6 (23)	13.67	14 (11)	37.21

The most popular two journals for both Arctic and Antarctic polynya research between 1980 and 2021 are the Journal of Geophysical Research-Oceans and Deep-Sea Research Part II-Topical Studies in Oceanography, which are affiliated with the WoS categories of oceanography. In addition, the Arctic polynya research community prefers the journals Polar Biology and Journal of Marine Systems, while the journals of Geophysical Research Letters and Antarctic Science are popular among Antarctic polynya researchers. Besides, except for the journals mentioned above, journals with high average citations per article also have a crucial influence in the field of polar polynya science, e.g., the Deep-Sea Research Part I-Oceanographic Research Papers and Marine Ecology Progress Series for the Arctic, and the Atmosphere-Ocean and Ocean Modeling for the Antarctic. Moreover, the WoSc of those journals in Table 5 confirms that studies related to oceanography, ecology, geoscience and multidisciplinary studies dominate polar polynya research.

### 3.3. International collaboration

Based on the 1,667 publications from 1980 to 2021, 4,478 authors from 45 countries/regions and 1,087 organizations comprise the polar polynya research community. The number of authors, organizations and countries involved in Arctic/Antarctic polynya studies is 2,302/2,145, 585/651, and 38/37, respectively. The greater number of organizations involved in Antarctic polynya research than in Arctic bodes well for more authors in the future.

Table 6 presents the performance of the top 20 productive countries in the study of polar polynya. Of the top 20 countries, 2 are from North America, 13 are from Europe, 3 are from Asia and 2 are from Oceania (a collective name for the islands scattered throughout most of the Pacific Ocean). An article may have a large number of authors from different countries. The United States (USA) has the leading place in polar polynya research with a predominant publication number of 631 (37.63%) and high citations per article (42.72), followed by Canada and Germany with 391 (23.32%) and 260 (15.50%) publications and also with high citations. Canada was the most productive country in the Arctic polynya research with 352 (40.51%) publications, followed by the USA and Germany. In addition, Canada, Germany, Norway, Denmark, Russia and Greenland have more publications on polynya studies in the Arctic than in the Antarctic. The USA, Australia, and the UK are the top three producers of Antarctic polynya research. Moreover, there are more publications on Antarctic polynya than Arctic polynya in the UK, Australia, Italy, South Korea and China. On the other hand, the USA, Japan, France, Sweden, Finland, Belgium, Netherlands and Poland have comparable publications in both polar regions.

**Table 6:** Top 20 productive countries in the study of polar polynya from 1980 to 2021 with respect to the average number of citations per article (TC/TP) and the proportion of international collaboration publications (ICP/TP) for both polar, Arctic and Antarctic, respectively.

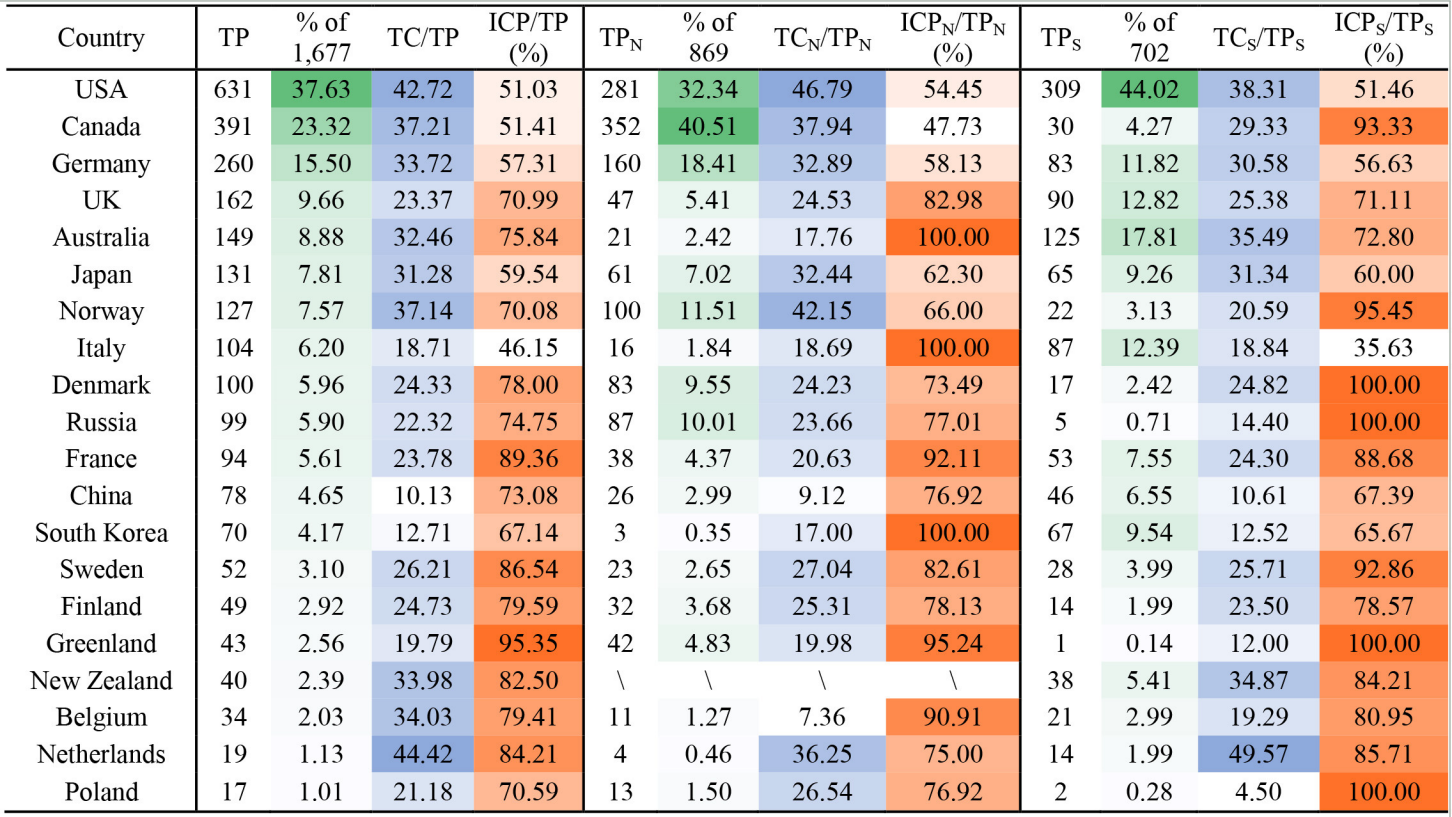

The dark color indicates high value, and vice versa.

**Figure 4:**
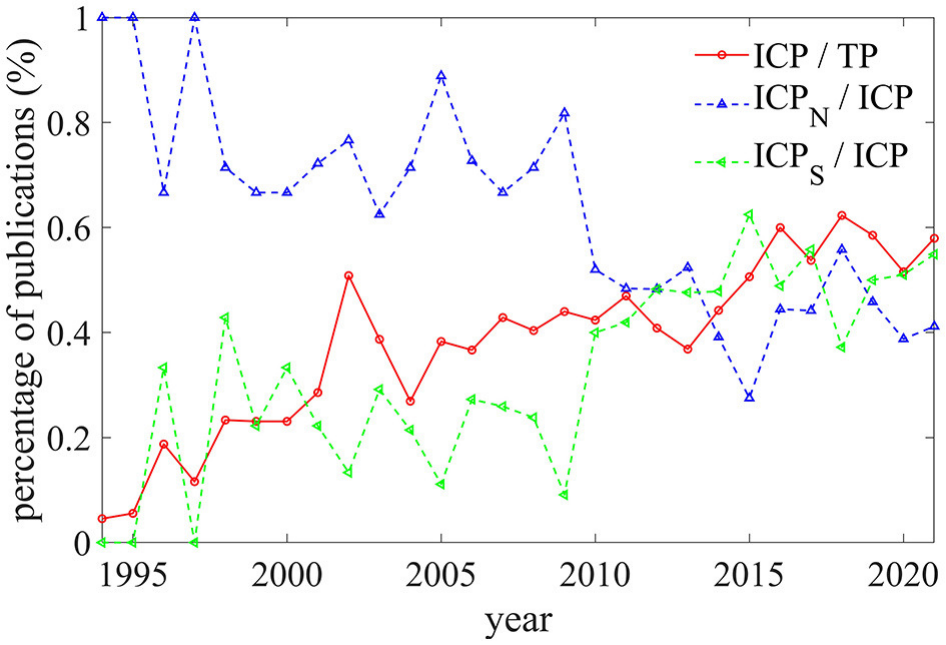
Percentage of international collaborative publications for both polar regions, the Arctic, and Antarctica, respectively, 1995–2021.

Figure 4 presents the temporal evolution of international cooperation. The polynya research field had very strong international collaborations with an average proportion of international articles (ICP/TP) of 72.14% in both polar regions, 78.42% in the Arctic, and 79.02% in the Antarctic. Extensive international cooperation in polar polynya research since about 1994 is presented in red in Figure 4. The two temporal phases, 1998 to 2002 and 2013 to 2016, present rapid growth of ICP. The ICP has exceeded 50% beyond 2015. The proportion of Arctic ICP (Figure 4, blue dotted line) has decreased along with the increase of Antarctic ICP proportion (green dotted line) which was partly attributed to the faster growth rate of publications on Antarctic publications.

**Figure 5:**
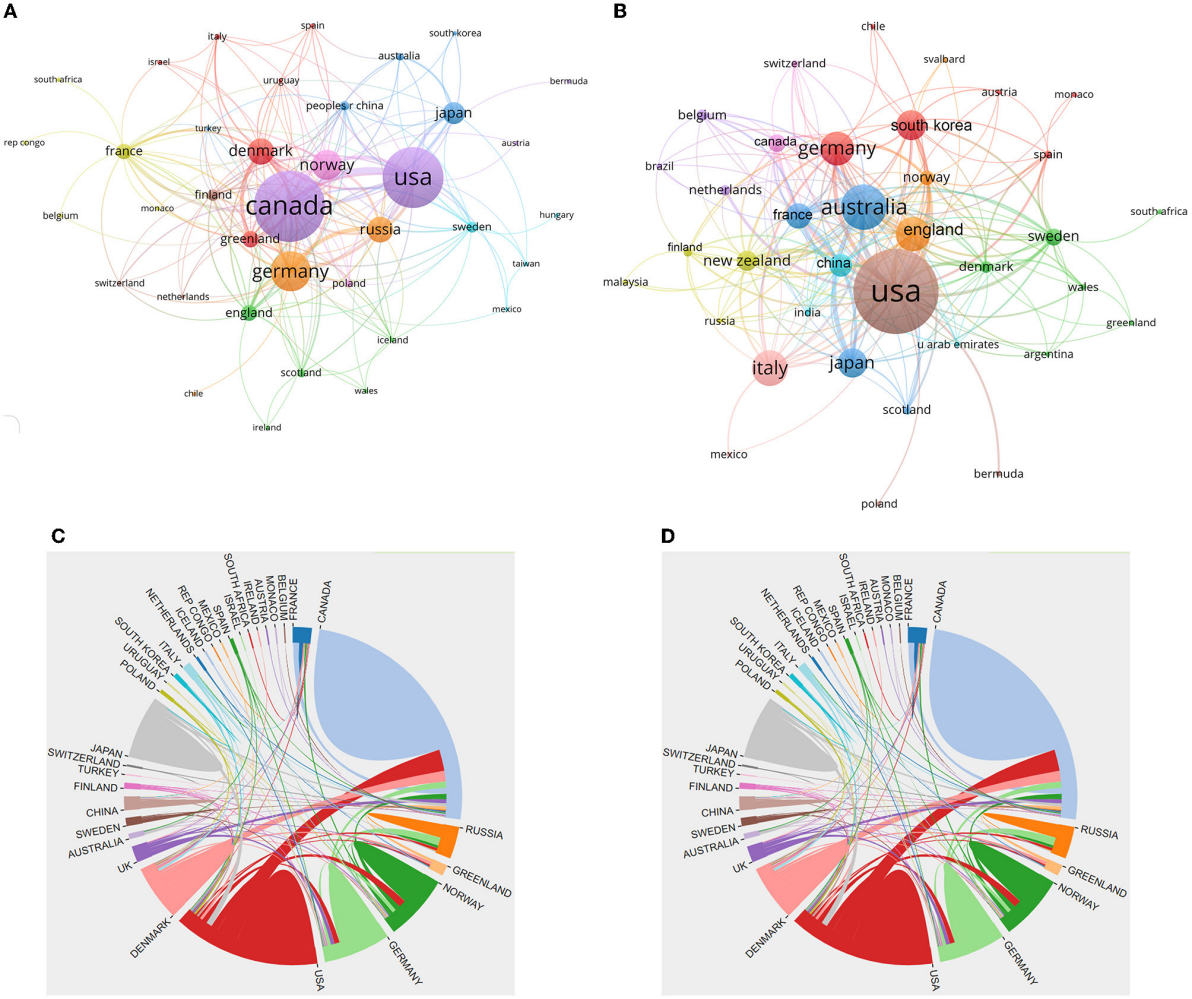
Collaboration network between countries [**(A, C)** for Arctic, **(B, D)** for Antarctic], 1980–2021. The size of circles in **(A, B)** indicates the number of publications.

Figures 5A, B give the collaboration relationships between countries associated with Arctic and Antarctic polynya studies, respectively. The Canada, USA, Germany, Denmark, and Norway comprise the top five international collaborations in Arctic polynya research. The USA, Australia, England, Germany, and France have been very cooperative in the study of the Antarctic polynya. Canada and the USA dominated research on polynya in the Arctic and Antarctic, respectively. The intensity of cooperation among countries shows that, while the United States has the most partners in the Antarctic polynya research field (Figure 5C), the highest intensity collaboration belongs to Australia and the United States, Australia and Japan, and Australia and France. On the other hand, Canada and the United States have archived higher-intensity collaborations in the field of Arctic polynya research (Figure 5D).

**Figure 6:**
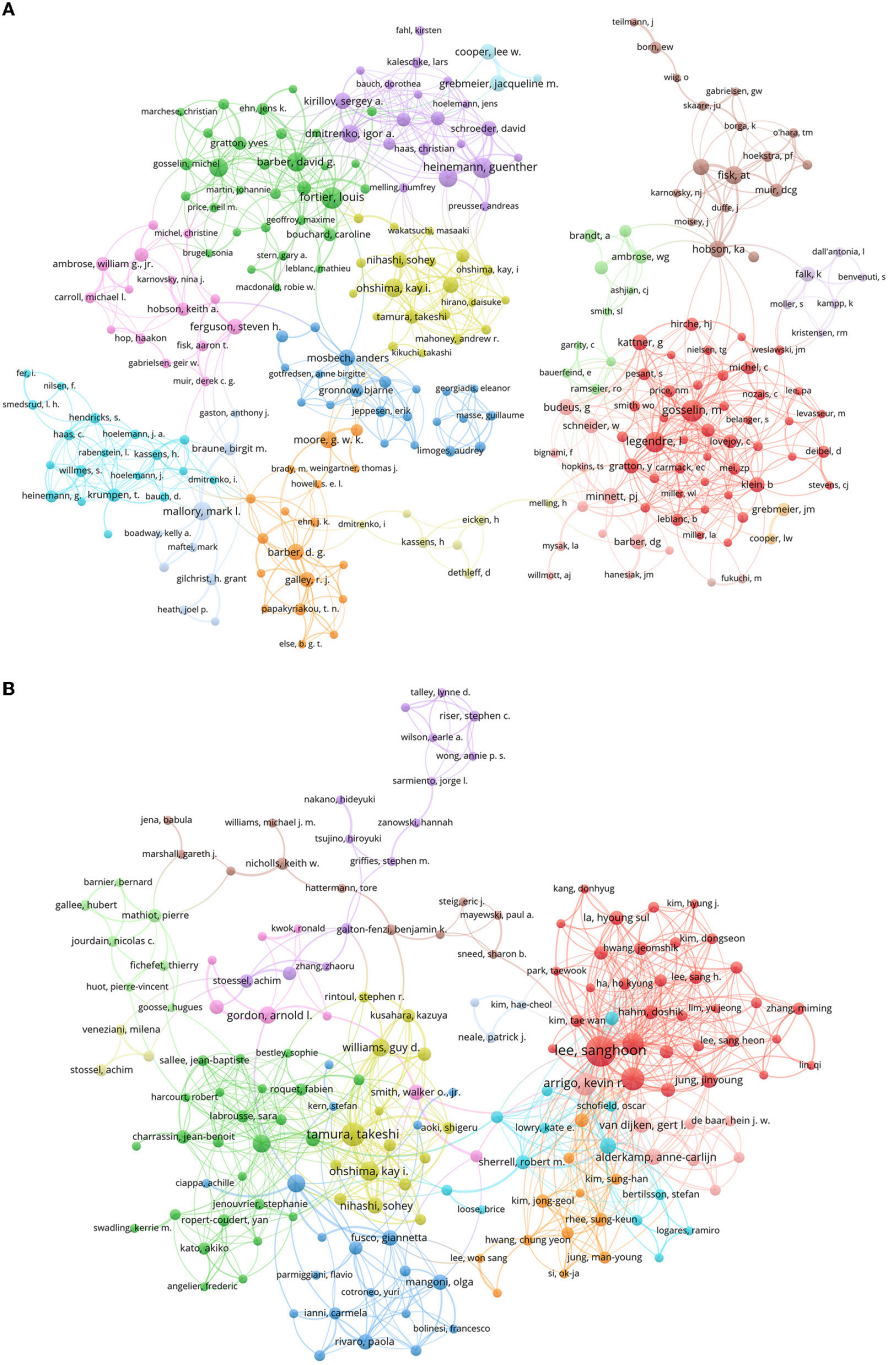
Clusters of authors for Arctic **(A)** and Antarctic **(B)**, 1980–2021. The size of circles indicates the weight of citations.

Figures 6A, B present the interactions between scholars in the field of Arctic and Antarctic polynya research with 17 and 13 clusters, respectively. For the Arctic, authors with the highest normalized citations in each cluster were Tremblay J.E., Moore G.W.K., Fisk A.T., Gosselin M., Barber, D.G., Mallory, M.L. (all from Canada), Heinemann G. and Krumpen T. (German), Grebmeier J.M. and Renaud, P.E. (USA), Ohshima, K.I. (Japan), etc. For the Antarctic, Tamura T. (Japan), Arrigo K.R., Gordon A.L., and Gordon A.L. and Stammerjohn S. E. (all from USA), Lee S.H. (Korea), and Fraser A.D. (Australia, Japan) were the most important authors in the main clusters.

### 3.4. Co-citation and bibliographic coupling analysis

**Figure 7:**
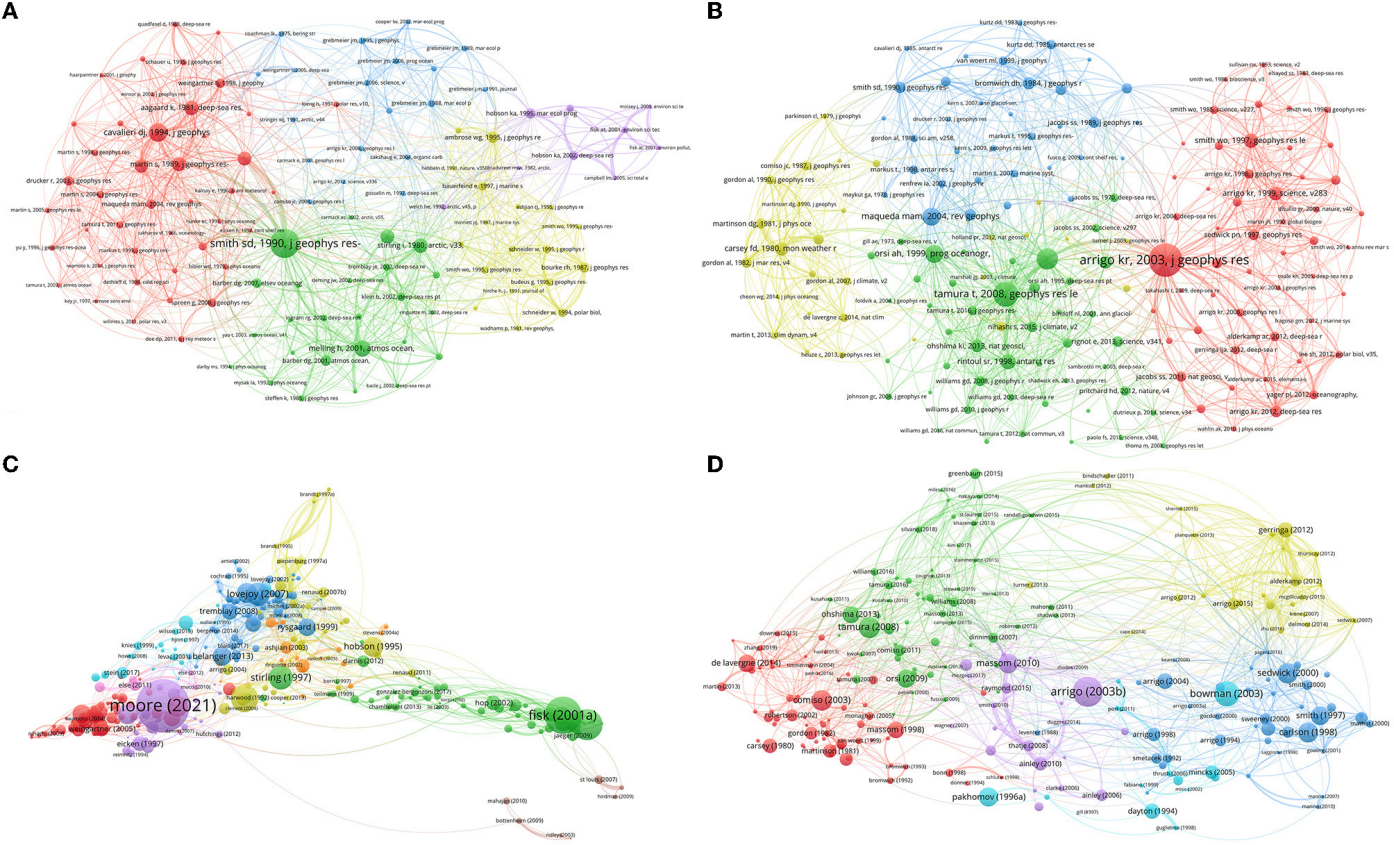
Clusters of co-cited references **(A, B)** and bibliographic documents **(C, D)** for Arctic **(A, C)** and Antarctic **(B, D)** polynya studies, respectively.

The co-citation analysis of citations shows the strength of the link within the cited references and the importance of the references. For the Arctic polynya study, out of 23,802 references with a minimum number of citations of 20, 118 references were obtained, resulting in 5 clusters as shown in Figure 7A. The most cited references for each cluster were “Polynyas and leads: An overview of physical processes and environment” (Smith et al., 1990; Comiso et al., 2008), “The contribution of Alaskan, Siberian, and Canadian coastal polynyas to the cold halocline layer of the Arctic Ocean” (Pease, 1987; Cavalieri and Martin, 1994), “Pelagic-benthic coupling on the shelf of the northern Bering and Chukchi Seas. I. Food supply source and benthic biomass” (Grebmeier et al., 1987; Comiso et al., 2008), “Benthic response to water column productivity patterns: Evidence for benthic-pelagic coupling in the Northeast Water Polynya” (Ambrose and Renaud, 1995; Hobson et al., 1995), and “Sources of primary production, benthic-pelagic coupling, and trophic relationships within the Northeast Water Polynya: insights from δ13C and δ15N analysis” (Hobson et al., 1995), respectively. The first two and fourth references belong to the area of oceanographic research, while the third and fifth are mainly concerned with environmental sciences and ecology. In addition, the most frequently cited reference, Smith et al. (1990), was partly attributed to the development of research on leads. For the South Pole, four clusters based on the 160 most popular references are obtained, as shown in Figure 7B. Among the most cited reference for each cluster are “Phytoplankton dynamics within 37 Antarctic coastal polynya systems” (Arrigo and van Dijken, 2003) about oceanography, “Mapping of sea ice production for Antarctic coastal polynyas” (Tamura et al., 2008) about geology, “Polynya dynamics: A review of observations and modeling” (Morales Maqueda et al., 2004) about geochemistry and geophysics, and “Microwave Observation of the Weddell Polynya” (Carsey, 1980) about meteorology and atmospheric sciences. The highest cited references highlight popular research directions in Antarctic polynya ecology. In short, Arctic polynya studies are mainly concerned with oceanography, and the study of Antarctic polynya involves more disciplines. Figures 7A, B also give insight into popular cited authors in this field.

In a bibliographic coupling analysis of documents, publications are divided into clusters based on shared references. Nine and seven clusters were obtained for the Arctic (Figure 7C) and Antarctic (Figure 7D) using documents with minimum citation counts of 30, respectively. The visualization of circles is based on the normalized number of citations received by a document. Those figures show the important and recently popular documents for each cluster. For example, in the Arctic (Figure 7C), the documents focusing on biogeochemistry were the more important publications (Fisk et al., 2001; Grebmeier et al., 2006; Lovejoy et al., 2007). The remarkable publication from Moore et al. (2021) reflects the recent hot research direction about the environment and climate change of sea ice. On the other hand, in the Antarctic (Figure 7D), the blue cluster was about research on Ross Sea polynya, which has no prominent publication in the past 10 years, as well as research on the marine community (cyan cluster) and ecology and biodiversity conservation (violet cluster). From about 2007 to 2015, studies on marine productivity were popular (yellow color) (Gerringa et al., 2012; Arrigo et al., 2015). In the most recent years, the research has mainly focused on the mass balance of ice shelves (green cluster) (Silvano et al., 2018; Stewart et al., 2019) and climate change in relation to ocean convection (red cluster) (de Lavergne et al., 2014; Zhang et al., 2019; Chang et al., 2020). The latter has a longer research history than the former.

### 3.5. Temporal evolution of Research Topics

In this section, we identify and compare polynya Research Topics for the Arctic and Antarctic regions, by investigating the frequency of keywords that reflect research hotspots and frontier regions. We focus the topic analysis on the remaining 1,621 publications after excluding 56 publications without keywords, most of which were published between 1980 and 1990.

Based on the keyword criterion claimed in Section 2.2, 3,614 keywords appeared 10,591 times in the 1,621 publications, and there were 2,268 keywords (5,556 times) and 1,704 keywords (4,419 times) for the Arctic and Antarctic polynya research, respectively. But most keywords (91.56% for all publications, 93.39% for the Arctic, and 92.84% for the Antarctic) occurred < 5 times. The dependence of the cumulant frequency on the keyword rank follows a power law distribution. Approximately 80% of keyword occurrences belong to the top 41.39% of publications for both polar regions, 51.01% for the Arctic and 48.18% for the Antarctic.

**Figure 8:**
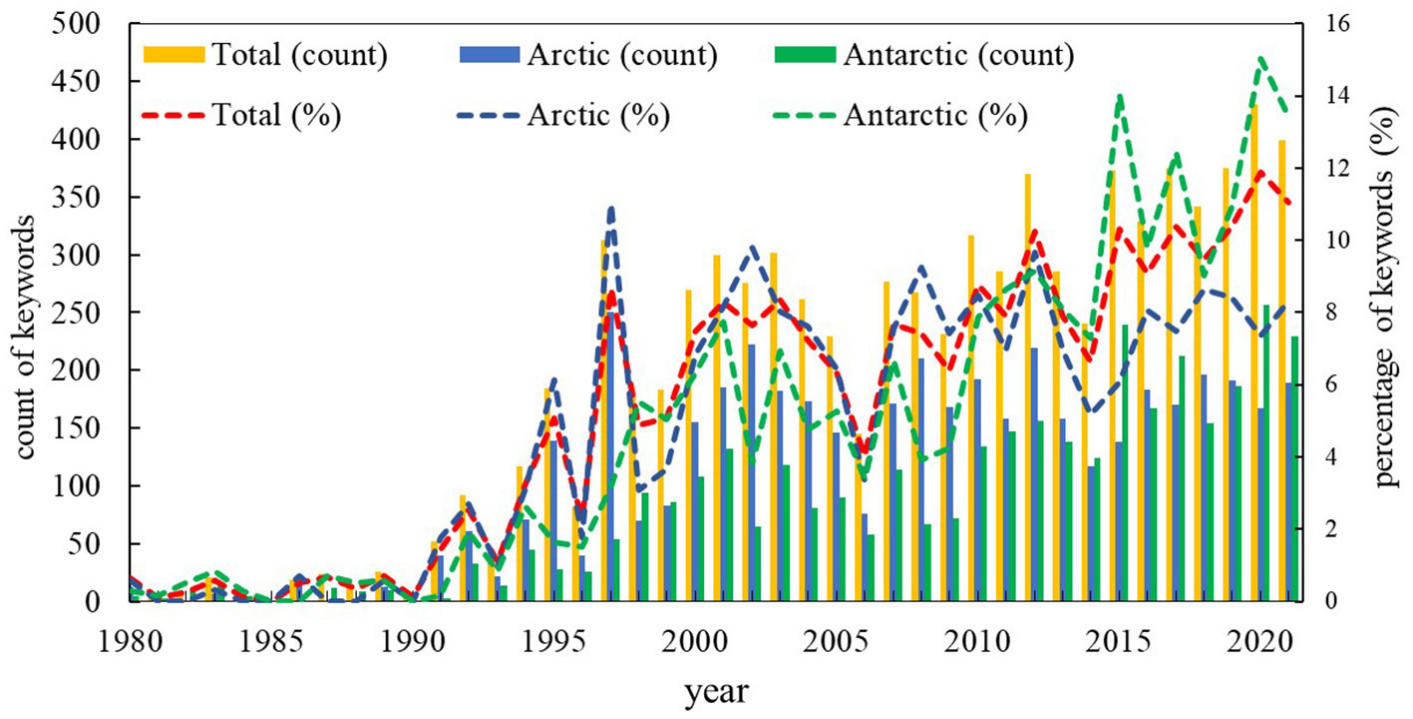
Count number and percentage of keywords by year for polynya publications on both polar (total), Arctic and the Antarctic, respectively, 1980–2021.

The annual count and percentage of keywords for both Arctic and Antarctic polynya studies show a growth tendency with years (Figure 8), which indicated a broader topic for polar polynya research. In the period 1991–2010, the keywords and publications on Arctic polynya study is approximately double that of Antarctic. Especially in 1997, which also resulted from a specific issue about Arctic polynya. In the last decade, the number of keywords appearing in Antarctic polynya research has surpassed that in the Arctic, which indicated the flourishing of Antarctic polynya research.

The frequently used keywords are given in Table 7 from 1991 to 2020 with a 10-year interval. In the three-decade phases, except for the keywords of the polynya, sea ice was the dominant keyword within the top 3 rank in both polar regions, followed by frequent keywords of variability, circulation, phytoplankton, model, climate change, and coastal polynya which ranked mainly within the top 25 and with increasing tendency. Keywords related to water were also popular in both polar regions in each decade.

**Table 7:** Counts and percentages of the top 25 most frequent keywords in the Arctic and Antarctic polynya studies from 1981 to 2020 with a 10-year time interval.

**Keywords (Arctic)**	**1991–2000**	**2001–2010**	**2011–2020**	**Keywords (Antarctic)**	**1991–2000**	**2001–2010**	**2011–2020**
**Polynya**	1 (46, 4.55)	3 (62, 2.72)	1 (51, 2.52)	**Polynya**	1 (14, 2.78)	1 (41, 3.64)	1 (75, 3.05)
**Sea ice**↓	3 (28, 2.77)	2 (65, 2.85)	1 (51, 2.52)	Ross sea↑	6 (9, 1.79)	2 (33, 2.93)	2 (65, 2.64)
Arctic ocean	2 (37, 3.66)	1 (69, 3.03)	3 (35, 1.73)	Southern ocean	3 (11, 2.18)	5 (23, 2.04)	3 (62, 2.52)
**Variability**↑	68 (2, 0.20)	13 (16, 0.70)	3 (35, 1.73)	**Sea ice**↑	3 (11, 2.18)	3 (29, 2.57)	3 (62, 2.52)
North water polynya	26 (4, 0.40)	4 (23, 1.01)	5 (26, 1.29)	Antarctica	1 (14, 2.78)	4 (28, 2.48)	5 (50, 2.03)
**Circulation**	11 (9, 0.89)	9 (19, 0.83)	6 (25, 1.24)	Amundsen sea↑	–	–	6 (39, 1.58)
Stable isotope	36 (3, 0.30)	17 (13, 0.57)	7 (20, 0.99)	**Variability**↑	42 (2, 0.40)	9 (12, 1.06)	7 (33, 1.34)
Remote sensing↑	26 (4, 0.40)	47 (6, 0.26)	8 (19, 0.94)	**Circulation**	70 (1, 0.20)	8 (15, 1.33)	8 (26, 1.06)
Phytoplankton	16 (6, 0.59)	7 (20, 0.88)	9 (18, 0.89)	Weddell sea	5 (10, 1.98)	5 (23, 2.04)	9 (25, 1.02)
Thickness↑	68 (2, 0.20)	31 (8, 0.35)	9 (18, 0.89)	Iron	70 (1, 0.20)	14 (6, 0.53)	10 (21, 0.85)
Chukchi sea	17 (6, 0.59)	23 (10, 0.44)	11 (16, 0.79)	Phytoplankton↑	23 (3, 0.60)	14 (6, 0.53)	11 (20, 0.81)
Bering sea	13 (8, 0.79)	13 (16, 0.70)	11 (16, 0.79)	Model↓	6 (9, 1.79)	7 (17, 1.51)	12 (18, 0.73)
Model↓	5 (15, 1.49)	10 (18, 0.79)	11 (16, 0.79)	**Climate change**↑	-	27 (4, 0.35)	13 (17, 0.69)
**Coastal polynya**	36 (3, 0.30)	16 (15, 0.66)	11 (16, 0.79)	East Antarctica↑	70 (1, 0.20)	14 (6, 0.53)	14 (16, 0.65)
Greenland	4 (19, 1.88)	14 (15, 0.66)	11 (16, 0.79)	**Coastal polynya**	10 (6, 1.19)	10 (8, 0.71)	14 (16, 0.65)
Baffin bay	23 (5, 0.50)	4 (26, 1.14)	16 (15, 0.74)	Circumpolar deep water↑	70 (1, 0.20)	152 (1, 0.09)	16 (15, 0.61)
Seabird↑	23 (5, 0.50)	31 (8, 0.35)	16 (15, 0.74)	Deep convection↑	42 (2, 0.40)	41 (3, 0.27)	17 (14, 0.57)
**Climate change**	–	20 (12, 0.53)	18 (14, 0.69)	Antarctic bottom water↑	70 (1, 0.20)	41 (3, 0.27)	17 (14, 0.57)
Beaufort sea	36 (3, 0.30)	13 (16, 0.70)	18 (14, 0.69)	Pine island glacier↑	–	–	17 (14, 0.57)
Halocline↑	37 (3, 0.30)	41 (7, 0.31)	18 (14, 0.69)	Shelf	23 (3, 0.60)	41 (3, 0.27)	17 (14, 0.57)
**Dynamics**	138 (1, 0.10)	22 (11, 0.48)	18 (14, 0.69)	Weddell polynya	23 (3, 0.60)	75 (2, 0.18)	21 (13, 0.53)
Mercury	-	12 (17, 0.75)	18 (14, 0.69)	**Dynamics**	70 (1, 0.20)	14 (6, 0.53)	21 (13, 0.53)
Water	14 (7, 0.69)	10 (18, 0.79)	23 (11, 0.54)	Katabatic wind	23 (3, 0.60)	41 (3, 0.27)	23 (12, 0.49)
Laptev sea	68 (2, 0.20)	23 (10, 0.44)	24 (10, 0.49)	Amundsen sea polynya	-	-	24 (11, 0.45)
Fresh water	138 (1, 0.10)	188 (2, 0.09)	25 (8, 0.40)	Impact	42 (2, 0.40)	41 (3, 0.27)	24 (11, 0.45)

The up arrow indicates increasing in importance. Keywords in bold show the common keywords of both polar. The data in the table represent rank (count, ratio). The shared keywords are highlighted in bold.

**Figure 9:**
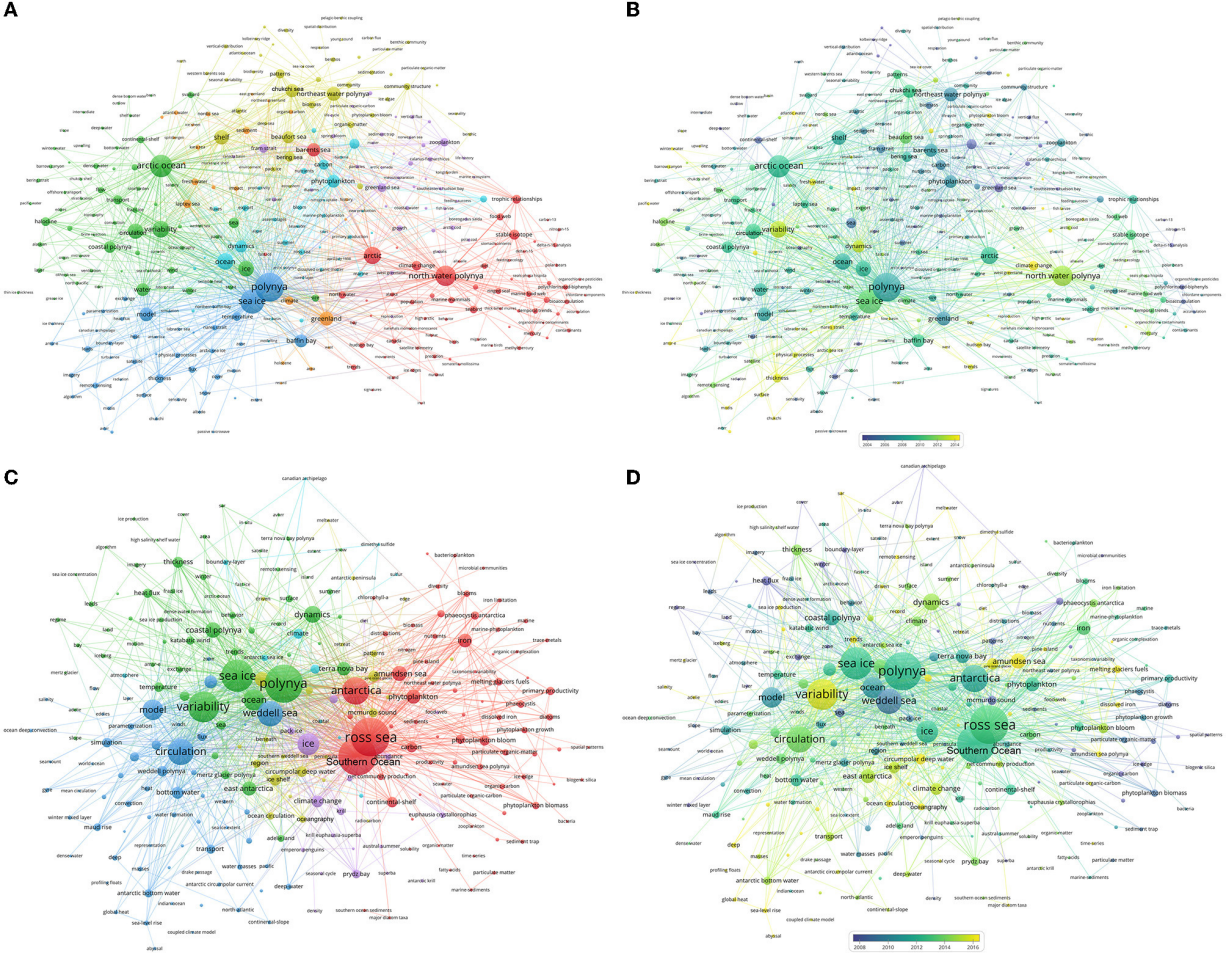
The cluster of the author keywords for the Arctic **(A)** and Antarctic **(B)** polynya research, overlay visualization map of the author keyword trends on the Arctic **(C)** and Antarctic **(D)** polynya.

The keywords clusters in Figure 9A reveal six Research Topics for Arctic polynya research: North Water Polynya (NOW) (red), Arctic ocean (green), sea ice (blue), Northeast Water Polynya (NEW) (yellow), zooplankton (purple), phytoplankton (cyan). The Arctic polynya was constantly focused on the NOW, located north of the Baffin Bay, which was highlighted by research on marine mammal and oceanography studies. Arctic ocean study in the field of polynya research was focused on circulation, halocline, dense/shelf water, entrainment, ventilation, flow, upwelling and so on. Sea ice was the most related item with polynya, in its cluster, researches were featured with model, temperature, thickness, flux and so on. The NEW located in eastern Greenland was the most popular polynya in the 1990s and 2000s, with numerous multidisciplinary publications focusing on sea ice and marine freshwater biology, which dwindled in the 2010s (Figure 9B). Zooplankton and phytoplankton, two divisions of Marine plankton biology, which developed in a smaller science community as a result of their heavily relies on field campaigns in harsh polar. In recent years, the most popular keywords for Arctic polynya were climate change (in the NOW cluster), thickness (in the sea ice cluster), ice production and variability (in the Arctic ocean cluster), Atlantic water (close to Arctic ocean cluster), and phytoplankton bloom (in the NEW cluster), which denoted the frontier of Arctic polynya research (Figure 9B).

There are six clusters derived from 256 items given in Figure 9C for the study of Antarctic polynya. For Ross Sea and Amundsen Sea (red), which are in the largest cluster, were highlighted with research about phytoplankton, iron, continental shelf and glacier. The sea ice cluster (green) in the southern ocean, which was focused on variability, dynamics, coastal polynya, heat flux and thickness, was similar to the Arctic. Polynya research about Weddell Sea (blue) was featured with keywords of circulation, model, water, bottom water, transport, bottom water, Maud Rise, deep convection and so on. On the other hand, research in pine island glacier (yellow) was more concerned about ice shelf, circumpolar deep water and ocean circulation. The minor cluster of ice (purple) liked the climate change, krill and prydz bay. Figure 9D shows that the Amundsen Sea, circumpolar deep water, ocean circulation, ice shelves, glaciers, katabatic winds, remote sensing, and atmosphere ocean interaction lead the current frontier of Antarctic polynya research. The Amundsen sea, which is one of the fastest melting areas in Antarctica (Jenkins et al., 2018), is the hotspot zone in the recent decade and is characterized by research on marine ecology and ocean water.

In the Ross Sea, keywords of Terra Nova polynya, ice production, heat flux, and fast ice (Figure 9C) were highly desirable in recent 20 years. More importantly, polynya studies related to ice shelves in East Antarctica have also been prevalent in recent years.

The frequently used keywords and aforementioned cooperation denote that, under the background of polar climate change, scholars tend to seek advanced technologies (e.g., remote sensing) and more cooperation (e.g., *in-situ* work) to acquire information about polynyas and understand their physical processes and their impacts on the cryosphere, especially in the active polynyas around the polar regions (Rabenstein et al., 2013; Hollands and Dierking, 2016; Aulicino et al., 2018).

## 4. Discussions and conclusion

In this study, a bibliometric investigation of scientific publications on polar polynya research was conducted based on the analysis of basic statistics of publications performance, the scientific categories of publications, popular journals, cooperation between countries, authors, co-cited references, bibliographic documents, and the evolution of keywords, with comparison of those aspects between the Arctic and Antarctic. This study reviewed 1,677 global scientific publications from the WoS core collection database, published between 1980 and 2021.

The results of this study reveal that the period from 1980 to 1990 was relatively quiet for the Arctic and Antarctic polynya research, then followed by a very active period beginning in 1991. The average annual increase in the number of publications is 28.37% and 15.30% for the Arctic and Antarctic from 1991 to 2021, respectively. An increase in the average number of citations and authors per publication followed. Those indicate that polynya research has received an increasing attention from scholars. However, the publication ratio of Arctic polynya in the overall Arctic scientific community is declining, while the opposite is true for Antarctic polynya (Figure 1B). The fluctuation in publications number may be partly attributed to the aperiodic *in-situ* work, which is the only way to study biological, hydrological, sedimentological and other physical processes associated with polynya, and the derivative international symposia (Deming et al., 1993; Hirche and Deming, 1997; Barber et al., 2001; Yager et al., 2012; Ackley et al., 2020). The differences between polynya studies in the two polar regions are highlighted by the prosperity of the Antarctic polynya and the slower growth of the Arctic polynya. This can be partly attributed to the heavy shift of the Antarctic ice sheet or glacier in recent years, which is associated with circumpolar polynya (Ciappa and Budillon, 2012; Criscitiello et al., 2013; Stewart et al., 2019; Liniger et al., 2020).

Between 1980 and 2021, the Arctic and Antarctic polynya were covered by 47 and 41 web science categories, respectively, with an annual trend of increasing numbers. Based on the categories and major journals, oceanography was the dominant scientific research category in polar polynya studies, with the environmental sciences, geosciences multidisciplinary, meteorology atmospheric sciences, remote sensing and other categories flourishing. This reflects the comprehensive and multidisciplinary nature of polar polynya research, indicating that systematic surveys and collaborations will assist in this field of science. On the other hand, Antarctic polynya publications in the first three categories had overtaken the Arctic. Between 1991 and 2021, the categories of ecology, environmental sciences, and geography and physical sciences ranked among the top three growth rates in the Arctic polynya research, with the category of marine freshwater biology experiencing the fastest decline. For the Antarctic in the same period, the fast growing categories belong to the geosciences multidisciplinary, imaging science and photographic technology, environmental sciences, and meteorology and atmospheric sciences. The Journal of Geophysical Research-Oceans and Deep-Sea Research Part II-Topical Studies in Oceanography was the top two most popular journals for both polar regions, while the Polar Biology and Geophysical Re-search Letters were the third most popular journals for the Arctic and Antarctic polynya, respectively.

In terms of international collaborations, between 1980 and 2021 there were 4,478 authors from 45 countries or regions and 1,087 organizations comprising the polar polynya research community. The USA occupies the dominant place with the most publications (37.63%) in both polar polynya and high citations per article (42.72), followed by Canada and Germany with 391 (23.32%) and 260 (15.50%) publications and also with high citations, respectively. Canada is the most productive country in the Arctic polynya research with 352 publications, followed by the USA and Germany. Australia is the second most significant country in the Antarctic polynya study (125 publications, 17.81%) after the USA (309 publications, 44.02%). Moreover, more and more non-polar countries have joined the scientific community of polynya study (e.g., Belgium, South Korea, and China), this reflects the increasing concern of polar environment from all over the world under the rapid changing polar regions (Anisimov et al., 2007). The percentage of international collaborative publications shows a growing trend toward Antarctic polynya, which has surpassed the Arctic in recent years.

The co-cited references show that Arctic polynya research is concentrated on oceanography, while Antarctic polynya research is multi-disciplinary. The bibliographic coupling document hints at the importance of biogeochemical studies as well as the emerging research on climate change related to Arctic sea ice. In the Antarctic, the popularity of research on Ross Sea polynya, marine communities, ecology and biodiversity conservation, and marine productivity is waning. Effects of polynya on mass balance of ice shelves and ocean convection points to the prevalence connection to climate change studies.

The Arctic polynya research mainly usually adopts keywords of “sea ice,” “North Water Polynya,” “variability,” “coastal polynya” and “phytoplankton,” and the Antarctic polynya research adopts “Ross Sea,” “sea ice,” “variability,” “Weddell Sea,” “circulation,” “model,” and “Amundsen sea.”

From a keyword evolution perspective, Arctic polynya studies have been directed toward investigating climate change, coastal polynya, thickness, Atlantic waters and phytoplankton bloom, with a growing number of publications on thicknesses, seabirds, and haloclines. For the Antarctic polynya, marine ecology and ocean water surveys in the Amundsen Sea have characterized hotspot zones, and together with ice shelves, remote sensing, and atmospheric-ocean interactions, have led the current frontier of Antarctic polynya research. Studies of ice production, heat flux, and fast ice in the Ross Sea occupy important places, followed by studies of water mass, ocean circulation, deep convection, and Antarctic bottom water in the Weddell Sea.

Polar polynya research is unique in that the content of the research is influenced not only by the scientific outcomes, but also by national policies in different countries. In addition, international field campaigns have had a significant impact on scientific research. Future research directions for polynya research are based on the results of existing studies.

Although this study provides a modern perspective on the current state of knowledge and trends in the field of polar polynya research, it has several limitations. The first deficiency comes from the incompleteness of the publication probabilities. Even though the search from Scopus yielded 1,659 publications under the same search strategy used for the WoS Core Collection database, publications from other platforms were ignored in this study. Secondly, the specific contents and current gaps of each research directions still need more work.

## Data availability statement

The original contributions presented in the study are included in the article/supplementary material, further inquiries can be directed to the corresponding author.

## Author contributions

TZ and FH had the idea for the article. TZ performed the literature search, data analysis, and drafted the work. HR, MS, FH, and XC critically revised the work. All authors contributed to the article and approved the submitted version.
